# Global Trends and Research Hotspots in Long COVID: A Bibliometric Analysis

**DOI:** 10.3390/ijerph19063742

**Published:** 2022-03-21

**Authors:** Hongxia Jin, Lu Lu, Haojun Fan

**Affiliations:** Institute of Disaster and Emergency Medicine, Tianjin University, Tianjin 300072, China; j714_star@tju.edu.cn (H.J.); lulu_998543@tju.edu.cn (L.L.)

**Keywords:** long COVID, bibliometric analysis, network analysis, research hotspots, research trends, publication, social network analysis

## Abstract

Long COVID is a condition distinguished by long-term sequelae that occur or persist after the convalescence period of COVID-19. During the COVID-19 pandemic, more and more people who tested positive for SARS-CoV-2 experienced long COVID, which attracted the attention of researchers. This study aims to assess the pattern of long COVID research literature, analyze the research topics, and provide insights on long COVID. In this study, we extracted 784 publications from Scopus in the field of long COVID. According to bibliometric analysis, it is found that: developed countries in Europe and America were in leading positions in terms of paper productivity and citations. The *International Journal of Environmental Research and Public Health* and the *Journal of Clinical Medicine* were leading journals in the perspective of publications count, and *Nature Medicine* had the highest number of citations. Author Greenhalgh T has the highest number of papers and citations. The main research topics were: pathophysiology, symptoms, treatment, and epidemiology. The causes of long COVID may be related to organ injury, inflammation, maladaptation of the angiotensin-converting enzyme 2 (ACE2) pathway, and mental factors. The symptoms are varied, including physical and psychological symptoms. Treatment options vary from person to person. Most patients developed at least one long-term symptom. Finally, we presented some possible research opportunities.

## 1. Introduction

Long COVID is a range of long-term sequelae after being infected with SARS-CoV-2. On 5 May 2020, Professor Paul Garner described that he felt constantly tired and dizzy after having COVID-19 [1]. Then, on 14 July, journalist Elisabeth Mahase ran a feature on what long COVID is [2]. Long COVID is a rapidly emerging research field after COVID-19.

Long COVID refers to a series of health consequences that are present four or more weeks after infection with SARS-CoV-2 [3,4,5,6], more specifically including two types: (1) ongoing symptomatic COVID-19 (symptoms last from 4 to 12 weeks) and (2) post-COVID-19 syndrome (symptoms last more than 12 weeks) [7,8]. Since the COVID-19 pandemic, numerous cases of long COVID have been identified [9], which has attracted the attention of researchers. This paper discusses major contributors and research hotspots of long COVID and provides references for future development.

The rapid production of knowledge makes it difficult for people to keep up with the latest research, so literature review becomes important [10]. In past studies, researchers from different countries noted long COVID: researchers from the United Kingdom described in detail how the phrase “long COVID” came to be [11]; researchers from the United States identified more than 50 long-term effects by systematic review and meta-analysis, and found that the most common effect was fatigue [9]; a prospective cohort study from Norway found that 61% home-isolated patients have long-term symptoms in the 6 months after infection SARS-CoV-2 [12]; a cohort study from China found that 6-month symptoms in patients discharged from hospitals were mainly fatigue, insomnia, anxiety, or depression [13]; a study from Israel reported long COVID cases of some patients infected with SARS-CoV-2 [14]. Long COVID has occurred in many countries, so we need the overview with an international perspective.

Long COVID affects people’s body health, such as muscle weakness, headache, and joint pain; meanwhile, it also has bad effects on people’s mental health, such as mood disorders, dysphoria, and obsessive compulsive disorder (OCD) [9]. In addition, because long COVID affects the insurance market, insurance companies need to investigate the characteristics of long COVID in more detail to develop appropriate insurance plans [15]; long COVID affects primary health care policy, so strengthening primary health care and addressing multifaceted inequalities are of great necessity [16]; long COVID influences health care and provides opportunities for the long-term care sector [17]. Since long COVID is closely related to clinical medicine, psychology, insurance, economics, management, nursing, and other fields, we need to make a comprehensive analysis.

This paper is based on previous research, uses knowledge graph software, and conducts a comprehensive and diverse analysis of literature related to long COVID. This study answers the following questions.

Q1: Which countries, journals, and authors are the main contributors to the research of long COVID? What kind of partnerships do they have?

Q2: What are the hot topics in long COVID?

## 2. Materials and Methods

### 2.1. Data

Data were obtained from Scopus for the following reasons:

(1) Scopus is an interdisciplinary database and 100% inclusive of MEDLINE;
(2) Scopus delivers the broadest abstract and citation database of peer-reviewed scientific literature;
(3) Scopus has advantages in the aspect of searching data, exporting literature, and analyzing citations.

**Figure 1:**
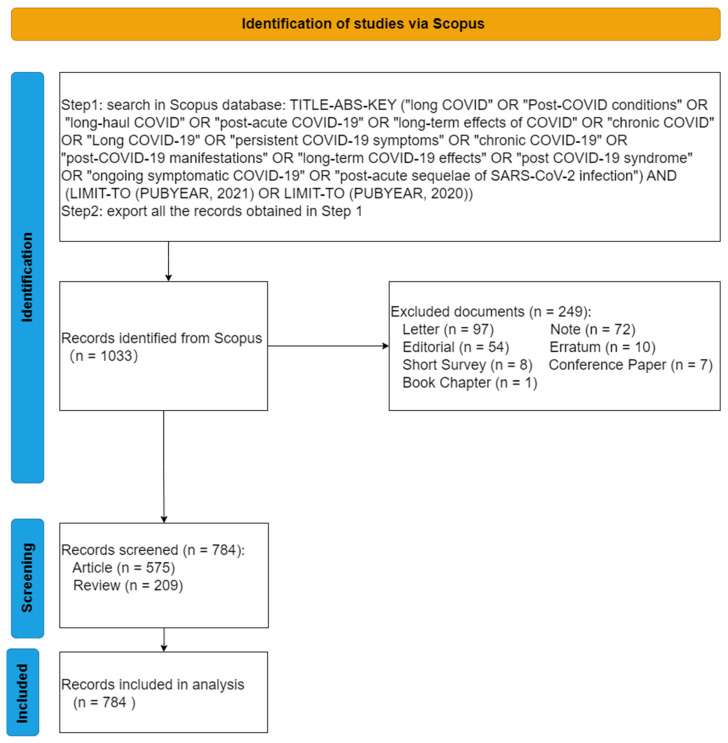
The flowchart of data selection.

The search strategy is shown in the flowchart (Figure 1) [6,7,8,9,11,18,19,20,21,22], and the publication years are 2020 and 2021. Two investigators independently searched on 5 January 2022. That the results were consistent indicated the search strategy was effective and repeatable. The time endpoint of the citations corresponded to the date of the extraction by the two investigators. We extracted 1033 publications from Scopus. When we only contained articles or reviews, 784 publications were included in this study. In the data process, we referred to the framework of the Preferred Reporting Items for Systematic reviews and Meta-Analyses (PRISMA) [23].

### 2.2. Methods

Bibliometric analysis is a quantitative research method for exploring publications topics and categories [24]. VOSviewer and Bibliometrix are both popular bibliometric tools: VOSviewer is a software that creates co-author maps or keywords co-occurrence maps based on literature data. Bibliometrix is an R-tool that provides bibliometric analysis of three different indicators (source, author, and document) and three knowledge structures (conceptual structure, knowledge structure, and social structure) [25].

The study was divided into two phases.

The first phase: we analyzed bibliometric indicators, which include leading countries, leading journals, and leading authors. We used Bibliometrix to draw country collaboration map as well as use VOSviewer to draw author co-authorship map and author co-citation map.

The second phase: we analyzed research topics. Through high-frequency keywords and keywords co-occurrence map, especially keywords co-occurrence map, we identified several research topics.

## 3. Results

**Figure 2:**
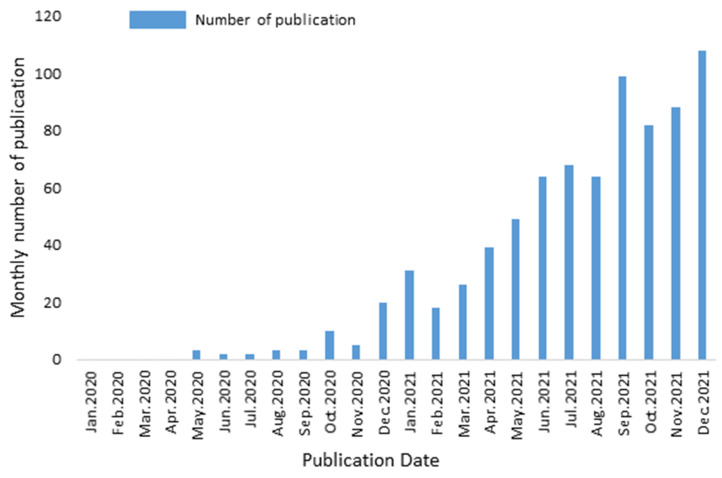
Monthly number of the publications.

The flowchart (Figure 1) illustrates the data process. In 2020, 48 publications were published, and the number of publications soared to 736 in 2021. Specifically, Figure 2 shows the monthly number of publications.

### 3.1. Leading Countries

According to the origin of the corresponding authors, a total of 61 countries and regions participated in the publications in this study. From the perspective of the number of publications, we counted the top 10 most active countries based on the corresponding authors’ country by Bibliometrix (Table 1). The United States produced the largest number of related publications (117, 14.9%), followed by the United Kingdom (74, 9.4%), Italy (71, 9.1%), Germany (46, 5.9%), and China (41, 5.2%). From the perspective of cited quantity, the United Kingdom was the most cited country (1027), and the average citations per publication was 13.88. The next was the United States (879), followed by Italy (275), Spain (192), and China (174). It was worth mentioning that the United States and the United Kingdom contributed 24.36% of the papers and 41.83% of the citations together.

**Figure 3:**
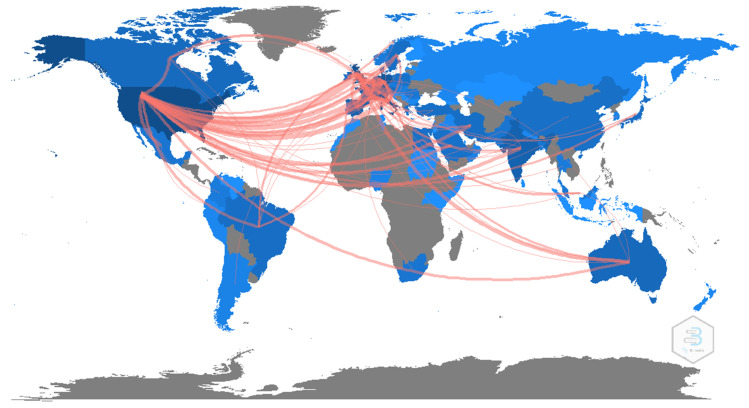
International research collaboration map in original articles on long COVID.

Figure 3 shows the degree of cooperation between participating countries. The blue color intensity on the map indicates the number of publications, and the thickness of the pink line indicates the degree of collaboration. The United States had large-scale cooperation with the European countries, especially the United Kingdom, Italy, and Germany. Meanwhile, there was also frequent cooperation among European countries.

### 3.2. Leading Journals

The 784 publications included in this study were published in 478 journals. From the perspective of the number of publications, we listed the top 10 most active journals (Table 2). The *International Journal of Environmental Research and Public Health* and the *Journal of Clinical Medicine* both had 20 publications, and *PloS ONE* was the next journal, with 14 publications. From the perspective of quoted quantity, *Nature Medicine* ranked number one, with four articles and 678 total citations. The *British Medical Journal* was the next, with 447 total citations. *Nature Medicine* and the *British Medical Journal* contributed 24.69% of citations.

### 3.3. Leading Authors

**Figure 4:**
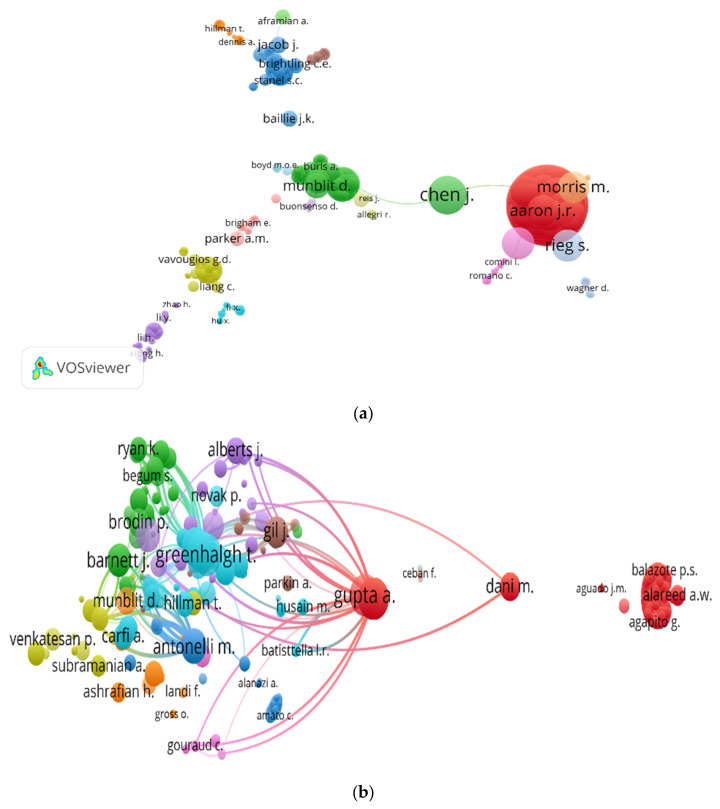
Network visualization map for (**a**) author co-authorship map; (**b**) author co-citation map.

There were 5747 authors in the 784 articles included in this study, with an average of 7.33 authors per article. Greenhalgh T published seven publications, Munblit D was the next leading author with six publications, followed by Sigfrid L, Tudoran C, and Tudoran M. An author co-authorship map and author co-citation map are shown in Figure 4a,b. In Figure 4a, Chen J had the largest total link strength (272) because he participated in two papers each with more than 50 authors [26,27]; in Figure 4b, Greenhalgh T and Gupta A both had the highest total local citations of 440 (i.e., how many times an author included in this field has been cited by the publications also included in the field).

### 3.4. Leading Articles

Table 3 lists the top 10 articles or reviews based on citations. The most cited publication was written by Nalbandian A et al., which summarized the pulmonary, hematologic, cardiovascular, neuropsychiatric, renal, endocrine, gastrointestinal, hepatobiliary, and dermatologic sequelae [6]; the second publication introduced how clinicians dealt with post-acute COVID-19; and the third publication reported the attributes and predictors of long COVID [28,29]. The total citations of all 784 publications was 4557, and the average citation number was 5.81. Among them, the top 10 publications had 1530 citations and contributed 33.57% of the total citations.

### 3.5. Research Hotspots

**Figure 5:**
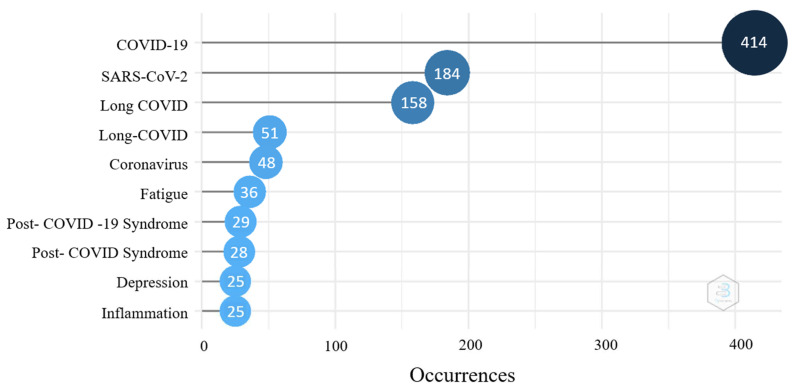
Top ten most frequent keywords.

Figure 5 demonstrates the 10 most frequent keywords. “COVID-19” was the most frequent keyword, followed by “SARS-CoV-2”. SARS-CoV-2 is a coronavirus and caused a disease, which is named COVID-19. SARS-CoV-2 and COVID-19 often appear together in a paper, so SARS-CoV-2 was the second most frequent keyword. Next were “long COVID” and “long-COVID”. “Fatigue” and “depression” were common physical and psychological symptoms, and their frequencies were 36 times and 25 times, respectively.

**Figure 6:**
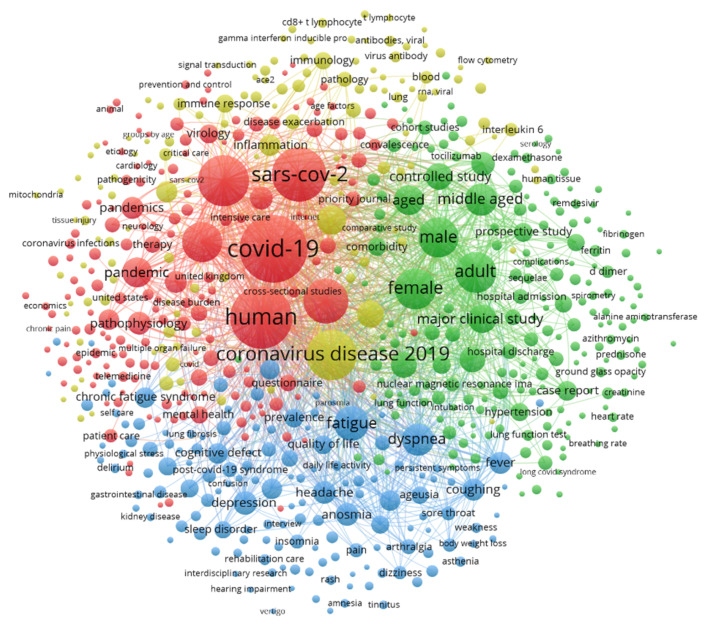
The keywords co-occurrence network.

A keywords co-occurrence network was generated (Figure 6). The size of each circle represents the frequency of keywords. The distance of each circle reflects the magnitude of relatedness of the keywords, and different colors of the circles represent different clusters.

In this study, we selected 642 keywords that appeared more than 5 times. Generally, four clusters were formed: pathophysiology (red group), symptoms (blue group), treatment (yellow group), and epidemiology (green group). These findings were critical because they helped us make sense of current research hotspots and provided references for exploring new research directions.

Red cluster: pathophysiology.

The pathogenesis of long COVID may be caused by several factors: (1) long-term damage to lungs, brain, heart, and other organs; (2) pathological inflammation (immune system dysregulation and hyperinflammatory state) [6,30]; (3) maladaptation of ACE2 pathway [6,31]; and (4) mental factors [30,32]. ACE2 is the receptor for SARS-CoV-2, and it plays an important role in the renin-angiotensin-aldosterone system, so maladaptation of the ACE2 might contribute to long COVID. Some studies compared long COVID with other diseases to explore its pathophysiology: one study suggested that the pathophysiology of post COVID-19 syndrome in the aspect of neurological symptoms may be similar to that of stroke [33]; another study compared long COVID patients (i.e., long hauler) with myalgic encephalomyelitis/chronic fatigue syndrome (ME/CFS) patients, which helped understand long COVID fatigue symptoms [34].

Blue cluster: symptoms.

The symptoms of long COVID were varied. First, there were some physical symptoms: fatigue [9,12,35], dyspnea [35,36], cough [35,36], loss of taste or smell [12], loss hair [9], fever [35,37], sleep disorder [38], joint pain [39,40], headache [41], spinal pain [42], muscle pain [39,43], diarrhea [44], and organ (heart, lungs, kidneys, liver, pancreas, and spleen) damage [45]. Second, there were some mental symptoms: insomnia, delirium, fear, and depression [46,47]. In August 2021, a research team found more than 50 symptoms, and in November 2021, another research group found over 100 symptoms [9,48]. Generally speaking, there were many kinds of symptoms.

Yellow cluster: treatment.

There was no standardized treatment for patients with long COVID. Instead, personalized treatment was recommended for each patient [30,49,50]. For specific symptoms, there were the following studies: (1) Robbins Tim et al. used hyperbaric oxygen therapy to treat chronic fatigue syndrome, which significantly improved fatigue [51]; Vollbracht C and Kraft K believed that injections of Vitamin C can help relieve symptoms of fatigue [52]; and (2) Dzubera A et al. used spinal surgery with radicular decompression to treat spinal pain in patients [42]. (3) Maria Ines Mitrani et al. found that amniotic fluid-derived extracellular vesicle biologic could be used to treat respiratory disorders [53]. (4) Najafloo R et al. proposed a comprehensive approach for the treatment of anosmia [54]. (5) Luckos M et al. used EEG neurofeedback to treat cognitive dysfunctions after long COVID-19 [55]. (6) Pilloni G et al. suggested that transcranial electrical brain stimulation (tES) could be used for mental health problems [56].

Green cluster: epidemiology.

Epidemiologists surveyed the patient groups and found that most patients developed at least one long-term symptom [6,9]. Women were more likely to develop long COVID than men; elderly people were more likely to develop long COVID than young people [57]; and existing comorbidity and BMI index were also associated with long COVID [58,59]. Children are a special group. A study of pediatric patients found that age, muscle pain at admission, and ICU admission were significantly associated with long COVID [60].

## 4. Discussion

### 4.1. Contribution of Countries, Journals, and authors

Developed countries in Europe and America were in a leading position in the field of long COVID in terms of the number of papers and citations. According to Jonathan P. Man, whether a paper can be published is related to research funds and English level [61]. Papers from English-speaking countries have a higher acceptance rate in high-level medical journals. According to Felicity Callard et al.’s article on the origin of long COVID, that COVID-19 patients shared their experiences on Twitter from March 2020 drew researchers’ attention to the long COVID [11,62]. European and American countries, especially Sweden, the United Kingdom, and the United States, had high average publication citations, which likely had relations with earlier median months of publication.

From the perspective of the number of articles, the topic of long COVID was not published in relatively centralized journals but was distributed in 478 journals. However, from the perspective of citations, *Nature Medicine* and the *British Medical Journal* performed best. According to Iman Tahamtan’s research, journals with a high impact factor are more likely to attract high-quality papers and help papers gain more recognition and citations [63]. The two journals also had earlier median months of publication, which likely helped get more citations.

On average, each paper included in the study had more than seven authors. Research by Sami Shaban et al. identified a trend toward multi-authorization in medical journals, which can be beneficial for collaboration, but may cause potential conflicts of interest among authors [64,65]. In Figure 4a, there are several clusters, which indicates there have been obvious expert groups. On the one hand, the formation of expert groups is conducive to sharing resources and accelerating the efficiency of communication; on the other hand, there may be a hidden danger of closure [66].

### 4.2. Analysis of Research Hotspots

In Figure 5, we listed the top ten most frequent keywords. “Fatigue”, “depression”, and “inflammation” were all in the lists. Although the frequencies of these keywords are high, it only means that these symptoms are common, and many authors discuss issues related to these symptoms. They were approximations of long-term COVID effects, which did not mean that these papers were reviews of long-term post-COVID effects or symptoms. In this case, the keyword co-occurrence map can better represent the research hotspots of long COVID.

Bibliometric analysis is a scientific method commonly used to explore new fields. Keywords co-occurrence analysis is a popular analysis method in bibliometric, which can effectively identify the structural characteristics of knowledge [67,68,69]. According to the keywords co-occurrence network, we found that the main research topics in the field of long COVID can be roughly divided into the following four aspects.

The pathophysiologic mechanisms of long COVID mainly included damage to lungs, pathological inflammation, maladaptation of ACE2, and mental factors. The symptoms of long COVID are varied, including physical and mental aspects. There is no standard treatment, differing from person to person. Most patients developed at least one long-term symptom. However, there are differences between men and women, old and young, and pre-existing medical conditions also play a role.

In the two years since the emergence of long COVID, a number of studies have been published. The current research predominant contained four topics, but the research did not stop there. For example: some researchers tried to define what “long COVID” is [21,70]; some researchers worked to diagnose long COVID [71]. It is foreseeable that more literature will be published in the near future, which will make our understanding of long COVID more comprehensive.

### 4.3. Research Opportunities

Although many researchers have devoted themselves to the study of long COVID, there are still some improvements to be explored due to the short period of occurrence of long COVID (about two years). We have presented some challenges and possible research opportunities.

Care plan.

There are many different types of symptoms of long COVID, so caring for patients is complex. For in-hospital and discharged patients, multidisciplinary experts need to work together to provide comprehensive care plans. Currently, it is urgent to establish a shared database that can enable healthcare professionals to identify, record, track, and manage patients’ conditions, as well as make clinicians identify the impact on health-related quality of life.

Other field effects of Long COVID.

There is currently a demand to learn more about the impacts of long COVID in other areas. (1) Education: the student patients may be absent from class for some time or convert from offline education to online education because of long COVID, so education experts need to examine whether these changes will affect students’ future academic development. (2) Economy: patients may need a long recovery period; therefore, economic experts need to explore the impact on residents’ income level, residents’ consumption level, stock market, and others. (3) Technology: long COVID promotes the improvement of nursing robots. It also promotes the progress of big data technology and artificial intelligence and the expansion of their application scope.

Special populations with long COVID.

Long COVID may have particular impacts on certain occupational or vulnerable populations. (1) Professional athletes. Doctors and coaches need to work together to monitor the status of athletes and adjust their training programs. (2) Weaker people: children, pregnant women, and old people. Weaker people are more susceptible, so we should investigate the effects of long COVID on them separately. (3) People with comorbidities. Researchers need to explore how long COVID affects these people.

### 4.4. Limitations

This study evaluated the trend of long COVID research through a bibliometric method, but there are some limitations at present. (1) Although we searched for long COVID-related keywords through a large number of high-level papers, we could not avoid missing relevant articles. (2) Due to the update of the Scopus database, the results are different with the same search keywords and different time periods, so relevant studies need to be updated in the future. (3) Because a potential length–time-effect bias exists, newer papers have disadvantages in citation, and the study may underestimate the influence of some papers.

## 5. Conclusions

This study is based on 784 articles or reviews from Scopus. We used bibliometric tools VOSViewer and Bibliometrix to analyze the development of long COVID. The results showed that developed countries in Europe and America were the most productive and cited regions, especially the United States and the United Kingdom. There was frequent cooperation between European and American countries. The *International Journal of Environmental Research and Public Health* and the *Journal of Clinical Medicine* were the most productive journals. *Nature Medicine* was the most cited journal. The most productive author was Greenhalgh T. The most cited authors were Greenhalgh T and Gupta A. In the field of long COVID, there have been obvious expert groups. By far the most cited article was “Post-acute COVID-19 Syndrome”.

Research topics were diverse, including pathophysiology, treatment, symptoms, epidemiology, health care policy, and public health management. The predominant pathophysiologic mechanisms include long-term organ damage, inflammation, maladaptation of ACE2 pathway, and mental factors. There were many kinds of symptoms including physical and psychological symptoms. There is no uniform standard of treatment, so doctors require to make individualized treatment projects for patients. Long COVID has a high incidence among COVID-19 patient. At present, there are still some issues to be addressed with long COVID, including influences on certain fields and special populations with long COVID, so further research is needed.

## Figures and Tables

**Table 1:** The top 10 most active countries based on the corresponding authors’ country.

Rank1 ^1^	Country	Number of Articles (%)	Rank2 ^2^	Country	Citations	APC ^3^	MMP ^4^
1	United States	117 (14.9%)	1	United Kingdom	1027	13.88	July 2021
2	United Kingdom	74 (9.4%)	2	United States	879	7.51	June 2021
3	Italy	71 (9.1%)	3	Italy	275	3.87	August 2021
4	Germany	46 (5.9%)	4	Spain	192	7.11	September 2021
5	China	41 (5.2%)	5	China	174	4.24	July 2021
6	Spain	27 (3.4%)	6	Sweden	154	25.67	April 2021
7	France	23 (2.9%)	7	Germany	139	3.02	September 2021
8	India	20 (2.6%)	8	India	78	3.90	July 2021
9	Australia	13 (1.7%)	9	France	76	3.30	October 2021
10	Canada	13 (1.7%)	10	Denmark	70	7.00	August 2021

^1^ Rank1: Ranking based on the number of articles; ^2^ Rank2: Ranking based on the number of citations; ^3^ APC: Average Publication Citations; ^4^ MMP: Median month of publication.

**Table 2:** The top 10 most active journals.

Rank1 ^1^	Journal	Number of Articles	Rank2 ^2^	Journal	Citations	MMP ^3^
1	*International Journal of Environmental Research and Public Health*	20	1	*Nature Medicine*	678	April 2021
2	*Journal of Clinical Medicine*	20	2	*British Medical Journal*	447	April 2021
3	*PloS ONE*	14	3	*Thorax*	135	April 2021
4	*Frontiers in Immunology*	11	4	*Journal of Infection*	126	July 2021
5	*Frontiers in Medicine*	11	5	*Acta Paediatrica*	118	July 2021
6	*Viruses*	10	6	*International Journal of Environmental Research and Public Health*	117	August 2021
7	*BMJ Open*	9	7	*Clinical Medicine, Journal of the Royal College of Physicians of London*	100	April 2021
8	*Journal of Medical Virology*	7	8	*BMJ Open*	91	August 2021
9	*Journal of Neurology*	7	9	*International Journal of Clinical Practice*	84	October 2021
10	*Frontiers in Psychiatry*	6	10	*European Journal of Nuclear Medicine and Molecular Imaging*	77	August 2021

^1^ Rank1: Ranking based on the number of articles; ^2^ Rank2: Ranking based on the number of citations; ^3^ MMP: Median month of publication.

**Table 3:** The top 10 articles or reviews based on citations.

Rank	Title	Author	PY ^1^	Citations
1	Post-acute COVID-19 syndrome	Nalbandian A	2021	396
2	Management of post-acute COVID-19 in primary care	Greenhalgh T	2020	365
3	Attributes and predictors of long COVID	Sudre C.H	2021	164
4	Long-COVID’: A cross-sectional study of persisting symptoms, biomarker and imaging abnormalities following hospitalisation for COVID-19	Mandal S	2021	135
5	Post-acute COVID-19 syndrome. Incidence and risk factors: A Mediterranean cohort study	Moreno-Pérez O	2021	109
6	Immune determinants of COVID-19 disease presentation and severity	Brodin P	2021	90
7	Autonomic dysfunction in ‘long COVID’: rationale, physiology and management strategies	Dani M	2021	84
8	Assessment and characterisation of post-COVID-19 manifestations	Kamal M	2021	68
9	Case report and systematic review suggest that children may experience similar long-term effects to adults after clinical COVID-19	Ludvigsson J.F	2021	60
10	How and why patients made Long COVID	Callard F.	2021	59

^1^ PY: Publication year.

## Data Availability

Search keywords, collect literature and use websites: https://www.scopus.com/ (accessed on 25 January 2022).
